# Hadamard Error-Correcting Codes and Their Application in Digital Watermarking

**DOI:** 10.3390/s24103062

**Published:** 2024-05-11

**Authors:** Michael Windisch, Jakob Wassermann, Monica Leba, Olimpiu Stoicuta

**Affiliations:** 1 Faculty Electronic Engineering & Entrepreneurship, University of Applied Sciences Technikum Wien, 1200 Vienna, Austria; jakob.wassermann@technikum-wien.at; 2 Automation, Computers, Electrical and Energy Engineering Department, University of Petrosani, 332006 Petrosani, Romania

**Keywords:** Hadamard matrix, 2D Hadamard transform, basis images, Enhanced Hadamard Code, Hamming distance, error-correcting capability, digital watermarking, visual light communication VLC

## Abstract

In communication technologies such as digital watermarking, wireless sensor networks (WSNs), and visual light communication (VLC), error-correcting codes are crucial. The Enhanced Hadamard Error-Correcting Code (EHC), which is based on 2D Hadamard Basis Images, is a novel error correction technique that is presented in this study. This technique is used to evaluate the effectiveness of the video watermarking scheme. Even with highly sophisticated embedding techniques, watermarks usually fail to resist such comprehensive attacks because of the extraordinarily high compression rate of approximately 1:200 that is frequently employed in video dissemination. It can only be used in conjunction with a sufficient error-correcting coding method. This study compares the efficacy of the well-known Reed–Solomon Code with this novel technique, the Enhanced Hadamard Error-Correcting Code (EHC), in maintaining watermarks in embedded videos. The main idea behind this newly created multidimensional Enhanced Hadamard Error-Correcting Code is to use a 1D Hadamard decoding approach on the 2D base pictures after they have been transformed into a collection of one-dimensional rows. Following that, the image is rebuilt, allowing for a more effective 2D decoding procedure. Using this technique, it is possible to exceed the theoretical error-correcting capacity threshold of ⌊$\frac{d_{min}-1}{2}$⌋ bits, where $d_{min}$ is the Hamming distance. It may be possible to achieve better results by converting the 2D EHC into a 3D format. The new Enhanced Hadamard Code is used in a video watermarking coding scheme to show its viability and efficacy. The original video is broken down using a multi-level interframe wavelet transform during the video watermarking embedding process. Low-pass filtering is applied to the video stream in order to extract a certain frequency range. The watermark is subsequently incorporated using this filtered section. Either the Reed–Solomon Correcting Code or the Enhanced Hadamard Code is used to protect the watermarks. The experimental results show that EHC far outperforms the RS Code and is very resilient against severe MPEG compression.

## 1. Introduction

In sensors applications such as wireless sensor networks (WSNs) or visible light communications (VLCs) the error-correcting codes play a very important role. For example, in wireless sensor networks, the data are transmitted from a field of distributed sensors, which monitors different parameters of the environment, for example, temperature, humidity, and wind, to the central sever, which collects and processes all these data. Signals can be falsified during the transmission, by bad weather conditions or some other obstacles, like a battery weakness of the sensors. Therefore, an efficient error-correcting code technique is required to secure the correct recovery of sensor data at the central server. In some publications, this problem has already been addressed [1,2,3]. The same problem occurs in visible light communications (VLCs), which is a part of optical communication. The transmission of information is realized using a light-emitting diode (LED) on the encoder side and a photodiode on the decoder side. By switching the light on and off, it is possible to transmit information. It is used mostly for short-distance communications, such as in hospitals, offices, and buildings, everywhere where the light is already used. The transmission of light should be flicker-free for human eyes and error-free and, therefore, RLL codes are combined with error-correcting codes. Some sufficient work in this area has been carried out in [4,5,6]. Generally, it can be said that error-correcting codes are a very important component of all sensor applications. In this paper, a new error-correcting code, the Enhanced Hadamard Code, is introduced, which could be sufficiently applied in the field of WSNs and VLC and could be easily installed there. The principle is explained and the efficiency in watermark technology is shown.

Many applications in the field of telecommunications use the Hadamard Error-Correcting Code. In 1960, Plotkin [7] made the initial discovery of the Hadamard matrix error correction capabilities. Bose and Shrikhande [8] and Peterson [9] have also contributed significantly. The Hadamard Error-Correcting Code construction algorithm was first developed by Levenshstein [10]. The NASA space mission of the Mariner and Voyager spacecraft in 1969 is the most well-known example of the use of the Hadamard Error-Correcting Code. The strong error-correcting ability of this code allowed for the accurate decoding of excellent images of Mars, Jupiter, Saturn, and Uranus [11]. The fundamentals of the Hadamard Code as well as a novel multidimensional Hadamard Code type known as the Enhanced Hadamard Error-Correcting Code (EHC) are presented in this study.

## 2. One-Dimensional Hadamard Error-Correcting Code

The Hadamard Error-Correcting Code was first introduced in 1969 by NASA in the Mariner and Voyager space missions to Mars. It has a very good error correction rate and a very simple decoding procedure. The Hadamard Code belongs to the special class of linear block codes generated via the Reed–Muller Code of the form $\left[2^{m},m+1,2^{m-1}\right]$. The $2^{m}$-bit long Hadamard Code can encode m+1 messages. The Hamming distance is $d_{min}=2^{m-1}$ and it can correct $\left\lfloor 2^{m-2}-\frac{1}{2}\right\rfloor$ errors. The error correction capability *t* of the Hadamard Code can also be calculated through the codeword length *n*. It is $\;t=\left\lfloor \frac{n}{4}-\frac{1}{2}\right\rfloor$.

During the Mars mission, the Marine 9 used the Hadamard Code [32,6,16] to transmit black and white pictures to Earth, and it was able to correct 7 bits. To understand the Hadamard Code in detail, the construction of the Hadamard matrices must be examined.

### 2.1. Hadamard Matrices

The Hadamard matrix $H_{n}$ of order n is defined as an n×n  matrix with elements 1 and −1. It fulfills the following equation:

(1) $$H_{n}\cdot H_{n}^{T}=H_{n}^{T}\cdot H_{n}=n{\cdot I}_{n}$$

where $I_{n}$ is an identity matrix.

To construct this type of matrix there are several approaches. The Sylvester Construction Method will be discussed here. For alternative methods and more details please refer to [12,13].

The Sylvester Construction Method is a recursive procedure for the generation of Hadamard matrices of order $2^{n}$. The procedure starts with a matrix $H_{1}=\left(1\right)$, and then generates other matrices through the following recursive algorithm:

(2) $$H_{2^{k}}=\left(\begin{array}{cc} H_{2^{k-1}} & {\;\;\;\;H}_{2^{k-1}}\\ H_{2^{k-1}} & {-H}_{2^{k-1}} \end{array}\right)$$

where 2≤k ∈ N.

Here is an example of the generation of the Hadamard matrix $H_{4}$:

$$H_{1}=\left(1\right)\;H_{2}=\left(\begin{array}{cc} 1 & \;1\\ 1 & -1 \end{array}\right)\;H_{4}=\left(\begin{array}{cccc} 1 & \;1 & \;1 & \;1\\ 1 & -1 & \;1 & -1\\ 1 & \;1 & -1 & -1\\ 1 & -1 & -1 & \;1 \end{array}\right)$$

It is important to mention here that there are other constructions of Hadamard matrices, such as Walsh–Paley matrices, but these are not used in the Hadamard Error-Correcting Code.

### 2.2. Generation of Hadamard Code

The n-bit one-dimensional Hadamard Code can be generated through the following steps:

Step 1: Generate an n×n Hadamard matrix $H_{n}$;.

Step 2: With the Hadamard matrix $H_{n}$, build a new matrix $C_{2n}$;

(3) $$C_{2n}=\left(\begin{array}{c} {\;H}_{n}\\ {-H}_{n} \end{array}\right)$$

Step 3: The rows of the matrix $C_{2n}$ are the codewords of the Hadamard Code and are denoted as ${\left\{{h_{i}|h}_{i}=C_{2n}\left(i,:\right)\right\}}_{i=1}^{i=2n}$; however, the −1 should be substituted with 0.

Example:

The generation of a Hadamard Code [8, 4, 4] is required. In the first step, a Hadamard matrix *H*_8_ is generated:

(4) $$H_{8}=\left(\begin{array}{cccccccc} \;1 & \;\;\;1 & \;\;\;1 & \;\;\;1 & \;\;\;1 & \;\;\;1 & \;\;\;1 & \;\;\;1\\ \;1 & -1 & \;\;\;1 & -1 & \;\;\;1 & -1 & \;\;\;1 & -1\\ 1 & \;\;\;1 & -1 & -1 & \;\;\;1 & \;\;\;1 & -1 & -1\\ 1 & -1 & -1 & \;\;\;1 & \;\;\;1 & -1 & -1 & \;\;\;1\\ 1 & \;\;\;1 & \;\;\;1 & \;\;\;1 & -1 & \;\;\;1 & -1 & -1\\ 1 & -1 & \;\;\;1 & -1 & -1 & -1 & -1 & \;\;\;1\\ 1 & \;\;\;1 & -1 & -1 & -1 & \;\;\;1 & \;\;\;1 & \;\;\;1\\ 1 & -1 & -1 & \;\;\;1 & -1 & -1 & \;\;\;1 & -1 \end{array}\right)$$

In the second step is the construction of the matrix:

(5) $$C_{16}=\left(\begin{array}{cccccccc} \;\;1 & \;\;\;1 & \;\;\;1 & \;\;\;1 & \;\;\;1 & \;\;\;1 & \;\;\;1 & \;\;\;1\\ \;\;\;1 & -1 & \;\;\;1 & -1 & \;\;\;1 & -1 & \;\;\;1 & -1\\ \;\;\;1 & \;\;\;1 & -1 & -1 & \;\;\;1 & \;\;\;1 & -1 & -1\\ \;\;\;1 & -1 & -1 & \;\;\;1 & \;\;\;1 & -1 & -1 & \;\;\;1\\ \;\;\;1 & \;\;\;1 & \;\;\;1 & \;\;\;1 & -1 & -1 & -1 & -1\\ \;\;\;1 & -1 & \;\;\;1 & -1 & -1 & \;\;\;1 & -1 & \;\;\;1\\ \;\;\;1 & \;\;\;1 & -1 & -1 & -1 & -1 & \;\;\;1 & \;\;\;1\\ \;\;\;1 & -1 & -1 & \;\;\;1 & -1 & \;\;\;1 & \;\;\;1 & -1\\ -1 & -1 & -1 & -1 & -1 & -1 & -1 & -1\\ -1 & \;\;\;1 & -1 & \;\;\;1 & -1 & \;\;\;1 & -1 & \;\;\;1\\ -1 & -1 & \;\;\;1 & \;\;\;1 & -1 & -1 & \;\;\;1 & \;\;\;1\\ -1 & \;\;\;1 & \;\;\;1 & -1 & -1 & \;\;\;1 & \;\;\;1 & -1\\ -1 & -1 & -1 & -1 & \;\;\;1 & \;\;\;1 & \;\;\;1 & \;\;\;1\\ -1 & \;\;\;1 & -1 & \;\;\;1 & \;\;\;1 & -1 & \;\;\;1 & -1\\ -1 & -1 & \;\;\;1 & \;\;\;1 & \;\;\;1 & \;\;\;1 & -1 & -1\\ -1 & \;\;\;1 & \;\;\;1 & -1 & \;\;\;1 & -1 & -1 & \;\;\;1 \end{array}\right)$$

Currently, the 16 rows of the matrix $C_{16}$, each containing an 8-bit codeword, define the Hadamard Code. The binary Hadamard Code [8,4,4] is displayed in Table 1, where 0 is used in the place of −1.

### 2.3. Decoding of Hadamard Code

In order to comprehend the decoding process, it is necessary to examine the codewords of the Hadamard Code. As previously stated, the rows of the Hadamard matrix $C_{2n}$, which are orthogonal to one another, are the codewords. A similar Hadamard spectrum can be constructed, represented by the *n*-dimensional vector $s_{i}$ by taking the *i*-th row, which is the n-dimensional codeword vector $h_{i}$.

(6) $$s_{i}=h_{i}\cdot H_{n}$$

Aside from component *i*, every element in this vector $s_{i}$ is zero. As a result, matching codewords can be decoded simply by looking at the received codewords’ Hadamard spectrum. The codeword is represented by the component of the spectrum vector with the highest value.

Take the following for instance:

Considering the Hadamard matrix $C_{2n}$, namely the third row (message word: 0010),

(7) $$h_{3}=\left[\begin{array}{cccccccc} 1 & 1 & -1 & -1 & 1 & 1 & -1 & -1 \end{array}\right]$$

the vector of the Hadamard spectrum is

(8) $$s_{3}=\left[\begin{array}{cccccccc} 1 & 1 & -1 & -1 & 1 & 1 & -1 & -1 \end{array}\right]\cdot H_{8}=\left[\begin{array}{cccccccc} 0 & 0 & 8 & 0 & 0 & 0 & 0 & 0 \end{array}\right]$$

The spectrum vector $s_{3}$ only differs from zero in its third component, namely

(9) $$s_{3}\left(3\right)=8,\;{\;\;s}_{3}\left(i\right)=0\;for\;all\;i\neq 3.$$

This indicates that the row in the Code Book (Table 2) is determined via the position $i_{3}$. The message word [0 0 1 0] is associated with the decoded codeword, which is located in the third row of the Hadamard matrix $C_{2n}$.The inverse row to the third row is row number eleven. The decoding process of this row delivers

$$s_{11}=\left[\begin{array}{cccccccc} -1 & -1 & 1 & 1 & -1 & -1 & 1 & 1 \end{array}\right]\cdot H_{8}=\left[\begin{array}{cccccccc} 0 & 0 & -8 & 0 & 0 & 0 & 0 & 0 \end{array}\right]$$

Also, in this case, the third component of the spectrum vector $s_{11}$ is different from zero. However, it is negative.

$$S_{11}\left(3\right)=-8,\;{\;s}_{3}\left(i\right)=0\;for\;all\;i\neq 3.$$

The decoding algorithm is defined as follows:

Step 1: Convert the codeword into the Hadamard vector by substituting the zeros with −1.

Step 2: Build the Hadamard spectrum using the Hadamard vector.

Step 3: Find the component with the largest absolute value. This component number defines the codeword.

Step 4: If the component value is negative, inverse the codeword.

In the example above, the third component has the largest absolute value $abs(s_{11}\left(3\right))=8$. The corresponding codeword is $h_{3}$. However, the spectrum value is negative ($s_{11}=-8)$, and, therefore, the codeword, $h_{3}$, must be inversed, which then becomes the codeword $h_{11}$.

If one error occurs inside the codeword $h_{3}$, the third component of the spectrum vector continues to have the highest value. Without restricting the generality, an error in the first component of the vector $h_{3}$ can be assumed:

(10) $$h_{3}=\left[\begin{array}{cccccccc} -1 & 1 & -1 & -1 & 1 & 1 & -1 & -1 \end{array}\right]$$

The decoding procedure delivers

(11) $$s_{3}=\left[\begin{array}{cccccccc} -1 & 1 & -1 & -1 & 1 & 1 & -1 & -1 \end{array}\right]\cdot H_{8}=\left[\begin{array}{cccccccc} -2 & -2 & 6 & -2 & -2 & -2 & -2 & -2 \end{array}\right]$$

The value of the third component of the Hadamard spectrum vector is still the greatest one. Despite the one error that occurs, it is still possible to decode the message word.

If there are two errors, it becomes difficult to decode the codewords with complete certainty. The following example illustrates this.

Consider a codeword with two errors,

(12) $$h_{3}=\left[\begin{array}{cccccccc} 1 & 1 & -1 & -1 & 1 & 1 & 1 & 1 \end{array}\right]$$

The two last components of the vector $h_{3}$ are flipped. The decoding procedure delivers

(13) $$s_{3}=\left[\begin{array}{cccccccc} 1 & 1 & -1 & -1 & 1 & 1 & 1 & 1 \end{array}\right]\cdot H_{8}=\left[\begin{array}{cccccccc} 4 & 0 & 4 & 0 & -4 & 0 & 4 & 0 \end{array}\right]$$

The components 1, 3, and 7 have the same value, namely

$$s_{3}\left(1\right)=s_{3}\left(3\right)=s_{3}(7)=4$$

Therefore, in this case (two errors), this codeword cannot be decoded unambiguously. Generally, the error-correcting capability *t* of any code with the Hamming distance $d_{min}$, can be calculated according to the formula

(14) $$t=\left\lfloor \frac{d_{min}-1}{2}\right\rfloor$$

In the case of the Hadamard Code [8,4,4] the Hamming distance is $d_{min}=4$ and, therefore, t=1. Another method for the calculation of the error-correcting capability of the Hadamard Code is

(15) $$t=\left\lfloor \frac{n}{4}-\frac{1}{2}\right\rfloor$$

where n is the length of the codeword.

The following conclusions apply:

- The Hadamard Code [8,4,4] is 8-bit long and can transmit 16 different messages. Only one error can be recovered unambiguously.
- General rule for the Hadamard Code: if the length of the Hadamard Code is n, then $\left\lfloor \frac{n}{4}-\frac{1}{2}\right\rfloor$ errors can be corrected. In the case of n=8, only one error can be corrected.

### 2.4. One-Dimensional Half-Mode Hadamard Code

Interesting results concerning error correction abilities can be achieved if only one Hadamard matrix generates the Hadamard Code.

Instead of using Equation (3) for the generation of the code, the following equation is used:

(16) $$C_{n}=H_{n}$$

In this case, we encode only *n* messages instead of 2*n* (see Table 2), but the number of correcting bits can be increased significantly. In addition to one error, now it is possible to correct seven or eight errors in one codeword.

The following example shows the case of 8-bit errors. This means, for example, that a Hadamard vector $h_{3}=\left[\begin{array}{cccccccc} 1 & 1 & -1 & -1 & 1 & 1 & -1 & -1 \end{array}\right]$ is completely corrupted and is received as ${\overset{`}{h}}_{3}=\left[\begin{array}{cccccccc} -1 & -1 & 1 & 1 & -1 & -1 & 1 & 1 \end{array}\right]$, and all bits are flipped (burst error). The same decoding procedure as before is now applied by building the Hadamard spectrum vector:

(17) $$s_{3}=\left[\begin{array}{cccccccc} -1 & -1 & 1 & 1 & -1 & -1 & 1 & 1 \end{array}\right]\cdot H_{8}=\left[\begin{array}{cccccccc} 0 & 0 & -8 & 0 & 0 & 0 & 0 & 0 \end{array}\right]$$

The component with the greatest absolute value defines the message word. In our case, ${abs(s}_{3}(3))=8$. According to Table 2, the corresponding message word of the third row is [0 1 0].

Without restricting the generality, in the case of 7-bit errors, the original Hadamard vector $h_{3}=\left[\begin{array}{cccccccc} 1 & 1 & -1 & -1 & 1 & 1 & -1 & -1 \end{array}\right]$ is corrupted and is received as ${\overset{`}{h}}_{3}=\left[\begin{array}{cccccccc} -1 & -1 & 1 & 1 & -1 & -1 & 1 & -1 \end{array}\right]$. The decoding procedure delivers

$$s_{3}=\left[\begin{array}{cccccccc} -1 & -1 & 1 & 1 & -1 & -1 & 1 & 1 \end{array}\right]\cdot H_{8}=\left[\begin{array}{cccccccc} -2 & 2 & -6 & -2 & 2 & -2 & -2 & 2 \end{array}\right]$$

and the component with the greatest absolute value defines the message word. Here, the third component has the greatest absolute value, ${abs(s}_{3}(3))=6$, which means that, according to the Code Book in Table 2, the received message word is [0 1 0].

In contrast to the full mode, only the absolute values of components are considered.

## 3. Two-Dimensional Hadamard Error-Correcting Code

The so-called Hadamard basis images can be used to extend the Hadamard Code. These basic images are utilized as codewords instead of Hadamard vectors.

By multiplying their columns and rows, the Hadamard matrix can be used to create the orthogonal basis image functions. The general guideline for creating a basic image is

(18) $$A_{lm}=H_{n}(:,l)\cdot H_{n}(m,:)$$

For example, in the case of a 4 × 4 Hadamard matrix,

(19) $$H_{4}=\left(\begin{array}{cccc} 1 & \;\;\;1 & \;\;1 & \;\;1\\ 1 & -1 & \;\;\;1 & -1\\ 1 & \;\;\;1 & -1 & -1\\ 1 & -1 & -1 & \;\;\;1 \end{array}\right)$$

A basis image $A_{31}$ is

(20) $$A_{31}=\left(\begin{array}{c} \;\;1\\ \;\;\;1\\ -1\\ -1 \end{array}\right)\cdot \left(\begin{array}{cccc} 1 & 1 & 1 & 1 \end{array}\right)=\left(\begin{array}{cccc} \;\;\;1 & \;\;\;1 & \;\;\;1 & \;\;1\\ \;\;\;1 & \;\;\;1 & \;\;\;1 & \;\;\;1\\ -1 & -1 & -1 & -1\\ -1 & -1 & -1 & -1 \end{array}\right)$$

**Figure 1:**
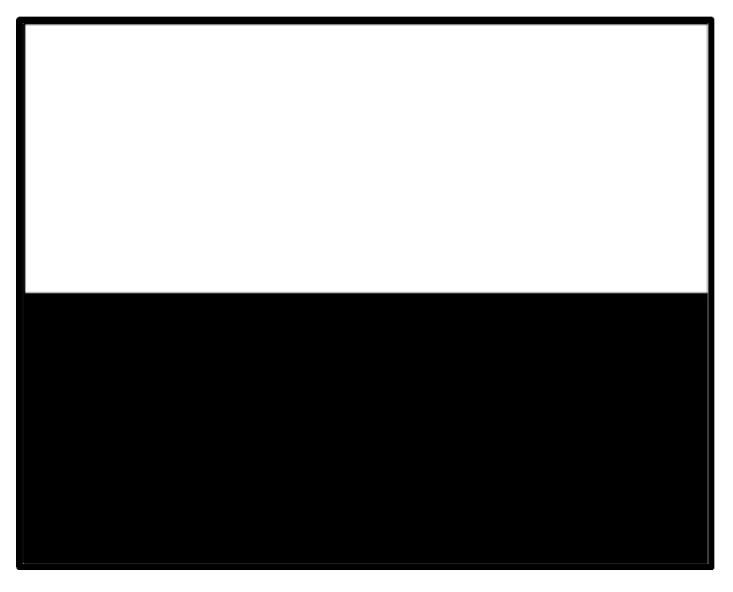
Basis Image $A_{31}$.

By replacing −1 with “black” and 1 with “white”, the basis image can be visualized, as depicted in Figure 1.

**Figure 2:**
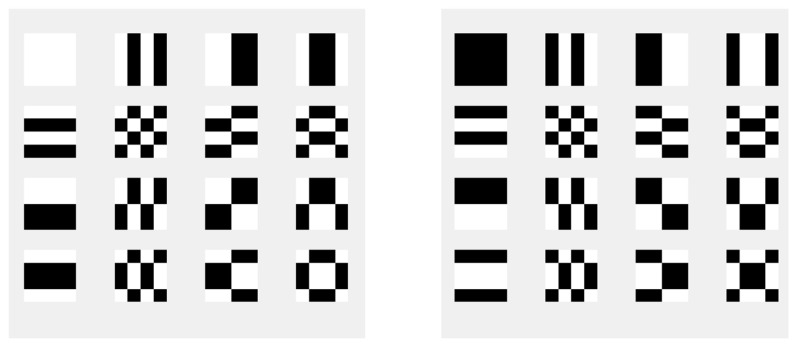
Basis Images of 4 × 4 Hadamard matrix.

A Hadamard matrix $H_{4}$ has thirty-two of these basis images. Figure 2 shows them all, as determined using Equation (18). It consists of two sets of sixteen basis images of Hadamard matrices, which are inverted versions of each other.

The 2D Hadamard spectrum of such basis images, indicated with *S*, is required for the decoding process. They can be computed using the formula

(21) $$S=H_{n}\cdot A_{lm}\cdot H_{n}^{T}$$

where $H_{n\;}$ is an n×n Hadamard matrix, $H_{n}^{T}$ is the transposed one, and $A_{lm}$ are the basis images. Since the transposed and the original matrices are equal, $H_{n}=H_{n}^{T}$, the calculation can be simplified.

The decoding process is identical to that of the one-dimensional scenario. The 2D spectrum, which is computed using Equation (21), is taken into consideration. The equivalent basis image is represented via the one non-zero element in the spectrum. This process is demonstrated in the example that follows. With basis image $A_{31}$ from Equation (20), the spectrum may be computed as follows:

(22) $$S=H_{4}\cdot A_{31}\cdot H_{4}=\left(\begin{array}{cccc} 0 & 0 & 0 & 0\\ 0 & 0 & 0 & 0\\ 16 & 0 & 0 & 0\\ 0 & 0 & 0 & 0 \end{array}\right)$$

The encoded basis image is defined by the location of the largest element in the matrix. S(3,1) = 16 in this instance; the remaining elements are all zeros. It indicates that the codeword is a basis image represented by a coefficient $A_{31}$. An error-correcting code may be built using the spectral coefficients to identify the basis images. According to Equation (21), the codewords, which are patterns of basis images, may be definitively deciphered by identifying the largest absolute coefficient value inside the Hadamard spectrum matrix. In [14], Wassermann and Dziech first introduced the corresponding basis images. They are mapped into a one-dimensional pulse stream by simply concatenating the image’s rows in order to create the codewords. The Code Book for the 2D Hadamard Code, which was created using 4 × 4 Hadamard matrix basis images, is displayed in Table 3. It is really a Hadamard Code with codewords that are 16 bits long, possessing a Hamming distance of $d_{min}=8\;\mathrm{bits}$. Three mistakes can be fixed by it. Ultimately, we obtain a standard one-dimensional Hadamard Code, but as we shall see later, the codewords can be interpreted as pictures, providing a fresh perspective on the code.

**Figure 3:**
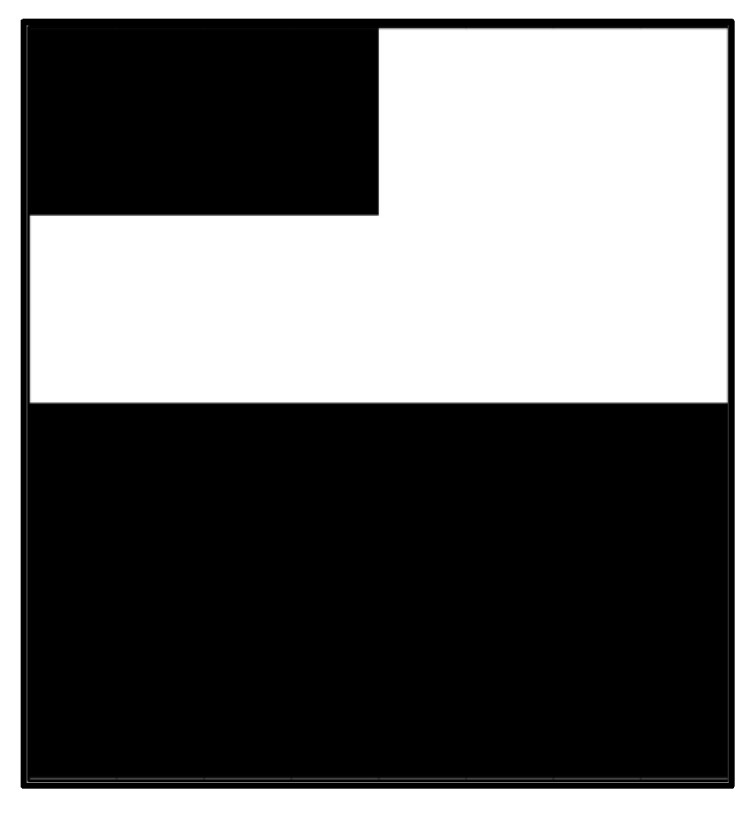
Corrupted Basis Image $A_{31}$.

Any corruption on the codewords automatically produces corrupted basis images. The analysis of codeword errors could be shifted to an analysis of distorted basis images. Figure 3 presents such a corrupted basis image. Any distortion affecting the codewords is transferred into the corresponding corruption of basis images.

If the number of errors does not exceed three, it is possible to completely recover the original pattern. This can be better understood by examining the following distorted basis image.

(23) $${\tilde{A}}_{31}=\left(\begin{array}{cccc} -1 & -1 & \;\;1 & \;1\\ \;\;\;1 & \;\;\;1 & \;\;\;1 & \;\;1\\ -1 & -1 & -1 & -1\\ -1 & -1 & -1 & -1 \end{array}\right)$$

The Hadamard spectrum, derived from Equation (21), is

(24) $$S=H_{4}\cdot {\tilde{A}}_{31}\cdot H_{4}=\left(\begin{array}{cccc} -2 & \;\;2 & -2 & \;\;2\\ -6 & -2 & -6 & -2\\ 10 & -2 & -6 & -2\\ -2 & \;\;\;2 & -2 & \;\;\;2 \end{array}\right)$$

The greatest spectrum component is S(3,1), which has an absolute value of 10. The associated message word may be read out from the Code Book and, according to Table 4, has the value (1 1 0 0 0).

## 4. Enhanced Hadamard Code

The Enhanced Hadamard Code provides the possibility to surpass the theoretical threshold of the error correction capacity of the Hadamard Code described in Equations (14) and (15). The first publication of this method was mentioned in [1]. This method was applied in video watermarking techniques.

As discussed earlier, the error correction of a code is determined by Hamming distance $d_{min}$, and the total number of errors that can be corrected is

(25) $$t=\left\lfloor \frac{d_{min}-1}{2}\right\rfloor$$

In the case of the Hadamard Code [16,5,8], an error-correcting capability of t=3 bit can be determined. With the Enhanced Hadamard Code, it is possible to correct more than three bits.

The fundamental concept behind the improved Hadamard Code is the mapping of 2D basis images into a set of 1D rows, which are then used in a 1D decoding process. Following this, the image is reassembled, making it possible to use the 2D decoding technique Equation (21) more effectively.

To show the functionality of the Enhanced Hadamard Code, we consider a Hadamard Code [64,7,32]. The codewords are 64-bit-long and they can encode $2^{7}=128$ different messages. The Hamming distance is $d_{min}$ = 32, which means that the code can correct 15 errors. These codewords can be interpreted via basis images of the size 8×8 of the 2D Hadamard transform. Without the restriction of any generality, we consider the basis image $A_{71}$.

(26) $$A_{71}=H_{8}\left(:,7\right)\cdot H_{8}\left(1,:\right)=\left(\begin{array}{cccccccc} \;\;\;1 & \;\;\;1 & \;\;\;1 & \;\;\;1 & \;\;\;1 & \;\;\;1 & \;\;\;1 & \;\;1\\ \;\;\;1 & \;\;\;1 & \;\;\;1 & \;\;\;1 & \;\;\;1 & \;\;\;1 & \;\;\;1 & \;\;\;1\\ -1 & -1 & -1 & -1 & -1 & -1 & -1 & -1\\ -1 & -1 & -1 & -1 & -1 & -1 & -1 & -1\\ -1 & -1 & -1 & -1 & -1 & -1 & -1 & -1\\ -1 & -1 & -1 & -1 & -1 & -1 & -1 & -1\\ \;\;\;1 & \;\;\;1 & \;\;\;1 & \;\;\;1 & \;\;\;1 & \;\;\;1 & \;\;\;1 & \;\;\;1\\ \;\;\;1 & \;\;\;1 & \;\;\;1 & \;\;\;1 & \;\;\;1 & \;\;\;1 & \;\;\;1 & \;\;\;1 \end{array}\right)$$

**Figure 4:**
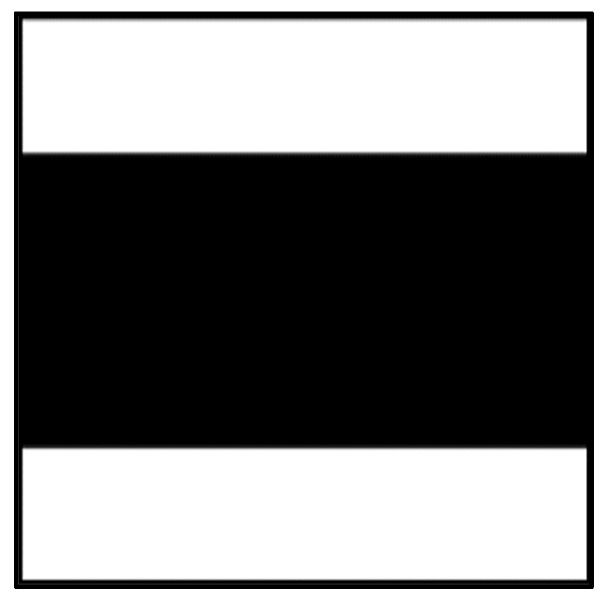
Basis Image *A*_71_.

The corresponding image is depicted in Figure 4.

**Figure 5:**
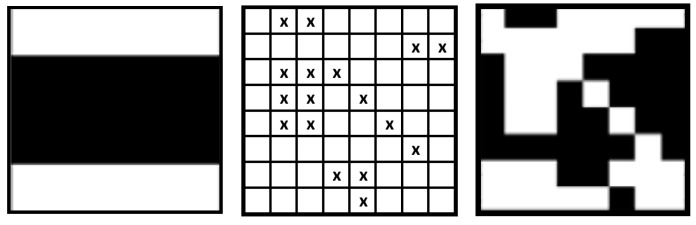
Original Basis Image *A*_41_, Error Mask, and Corrupted Pattern.

Figure 5 shows an image that has been distorted by noise. The error matrix in question comprises a total of 17 errors, which surpasses the predetermined threshold of 15 errors.

**Figure 6:**
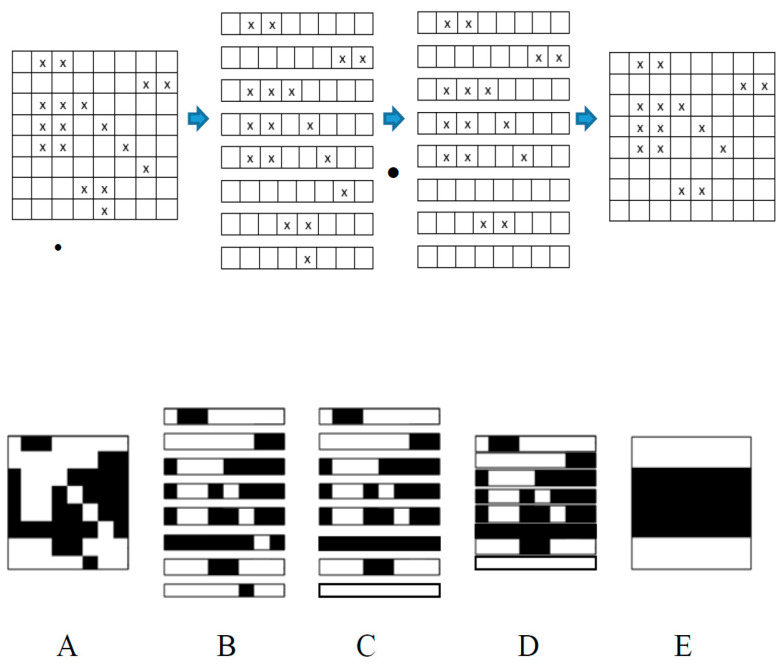
Enhanced Hadamard Decoding Procedure on Error Mask and on Corrupted Basis Image.

Figure 6 illustrates the operational capabilities of the improved Hadamard decoding technique.

The algorithm’s stages can be explained as follows:

∘ The tainted basis image (A) is broken down into its individual rows (B).
∘ The 1D Hadamard decoding process is executed to every row. Rows that contain only one error are decoded error-free; all their errors are removed. In the example, these are rows No. 6 and 8. In Part (C), these rows are depicted error-free.
∘ The rows are reassembled (D). There are 15 fewer mistakes in the updated pattern than there were in the previous one.
∘ The Hadamard decoding procedure is applied according to Equation (21) and the Code Book from Table 3. An error-free pattern is the end product: (E).

**Figure 7:**
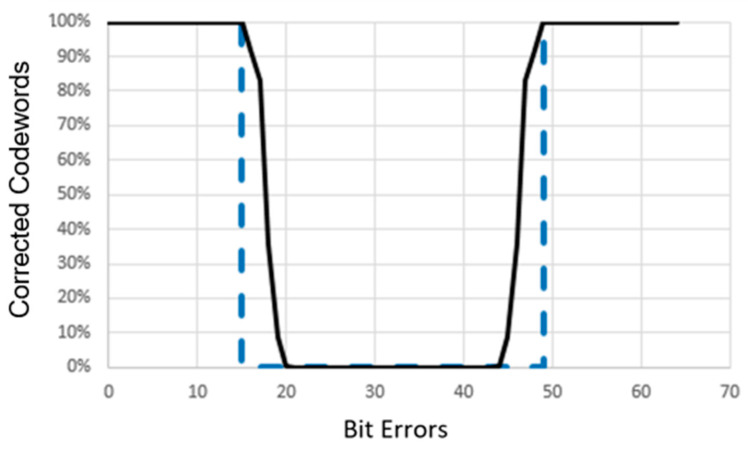
Comparison of error-correcting capabilities between the Standard Hadamard Code (shown with the dashed line) and the 8 × 8 2D Enhanced Hadamard Code.

A simulation is conducted to evaluate the error correction capabilities of the improved and standard Hadamard Error-Correcting Codes using codewords of length n = 64. Figure 7 illustrates the results. The *x*-axis represents the amount of bit mistakes, while the *y*-axis represents the proportion of repaired codewords. The improved 2D Hadamard Code is shown with a solid line, whereas the conventional 2D Hadamard Code is represented with a dashed line. The standard Hadamard Error-Correcting Code, as explained in Section 2.1, possesses the following characteristics: If the number of mistake bits falls between the range of [1, n/4 − 1] to [3n/4 + 1, n], this system is capable of completely correcting all erroneous codewords of length n, achieving a 100% success rate. If n = 64, the conventional Hadamard Code is capable of correcting mistakes when their quantity falls between the ranges of 1 to 15 and 49 to 64. With the Enhanced Hadamard Correcting Code, errors that exceed these limitations can be fixed. In the instance of 16 mistakes, 92% of all potential error patterns inside the codeword can be corrected. Even with 17 faults, there is still a possibility to rectify 83% of all error patterns. In the case of the Standard Hadamard Code, no mistakes can be rectified when there are 48 errors. However, the Enhanced Hadamard Code is capable of correcting faults in this scenario. It has the ability to rectify 92% of mistake patterns.

### Enhanced 3D Hadamard Error-Correcting Code

Diluting the Enhanced Hadamard Code into three dimensions was first mentioned in [14], and this can enhance the performance of the Hadamard Code. Instead of utilizing basis images, basis cubes (extensions of basis images) might be employed to create a comprehensive Code Book.

A Hadamard cube is created by extending a basis image into the third dimension by the multiplication of the basis image pattern with Hadamard vectors.

(27) $$D_{mlk}=A_{ml}\cdot H_{k}(:)$$

The basis image is denoted as $A_{ml}$, while $H_{k}(:)$ represents the k Hadamard vector. As an illustration, consider the pattern $A_{41}$.

$$A_{41}=\left(\begin{array}{cccc} 1 & -1 & -1 & 1\\ 1 & -1 & -1 & 1\\ 1 & -1 & -1 & 1\\ 1 & -1 & -1 & 1 \end{array}\right)$$

**Figure 8:**
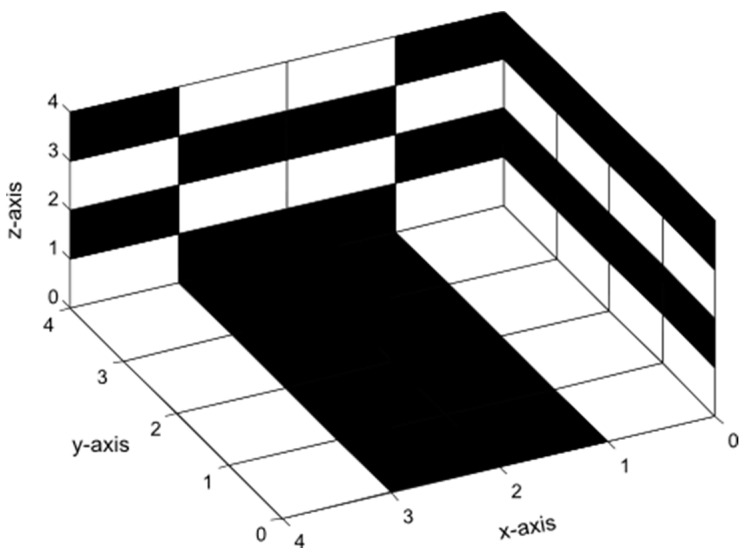
Hadamard Cube D_412_.

With the help of the corresponding Hadamard vector $H_{2}=[1-1\;1-1]$, the generation of the cube *D*_412_ could be realized, as shown in Figure 8.

**Figure 9:**
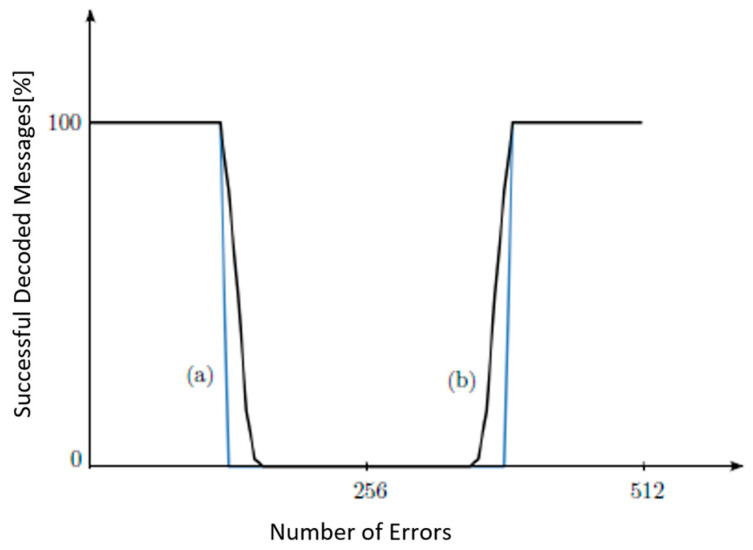
Comparison of error-correcting capabilities between the Standard Hadamard Code (a—blue line) and the 3D Enhanced Hadamard Code (b—black line).

The decoding process and the related error correction both function in a similar manner to the correction process outlined in Section 2. Prior to application, the cube is disassembled layer by layer starting from the front side. The upgraded 2D Hadamard decoding algorithm is used on each layer. A simulation was conducted to evaluate and compare the performance of the 3D Hadamard Code with the Standard Hadamard Code of length n = 512. The cubes have dimensions of 8 units in length, 8 units in width, and 8 units in height. Figure 9 displays the results.

## 5. Utilization of Enhanced Hadamard Code in Watermarking Technology and Its Outcomes

Digital watermarking is a promising emerging technique that presents numerous potential applications [15]. The issue of safeguarding intellectual property rights for audiovisual data from unauthorized use or tampering can be effectively addressed with the implementation of watermarking technology [16]. An essential element is an error-correcting code. The error-correcting algorithm employed in the watermarking technique is crucial for the survival of watermarks, particularly when watermarked video sequences are subjected to intense compression. These strategies are chosen to demonstrate the efficacy of an Enhanced Hadamard Error-Correcting Code (EHC). In order to highlight the effectiveness of EHC, it was compared to the widely recognized Reed–Solomon Code [17], itself employed in the identical watermarking scheme.

### 5.1. Proposed Scheme for Embedding Watermarks

**Figure 10:**
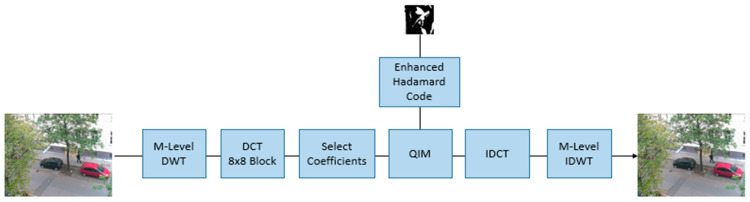
Watermarking Encoding Procedure.

The suggested watermarking approach operates in the spectral domain and employs an interframe discrete wavelet transform (DWT) on video sequences [14,18], together with an intraframe discrete cosine transform (DCT) for the embedding process [19,20]. Figure 10 provides a comprehensive illustration of the entire encoding process. The original video stream’s luminance channel is deconstructed using a multi-level interframe DWT with the Haar wavelet, resulting in a raw format. The video stream is subjected to a block-wise discrete cosine transform (DCT) after being filtered with a low-pass filter. The DCT spectrum is utilized to determine specific coefficients that are then employed in the embedding process with 3D Hadamard-coded watermarks. The embedding mechanism itself is implemented using Quadrature Index Modulation (QIM) techniques [21,22].

**Figure 11:**
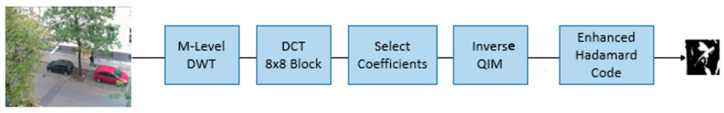
Watermarking Decoding Process.

The process of decoding is illustrated in Figure 11. During the decoding process, the embedded video sequence passes through the same multi-level interframe discrete wavelet transform (DWT) and intraframe discrete cosine transform (DCT) as it did during the encoding process.

Once the appropriate DCT coefficients have been chosen, the inverse QIM (IQIM) is implemented. The system transmits the deciphered codewords in the form of a pulse stream. The original watermark is extracted using the Enhanced Hadamard Error-Correcting Code.

#### 5.1.1. Multi-Level DWT

**Figure 12:**
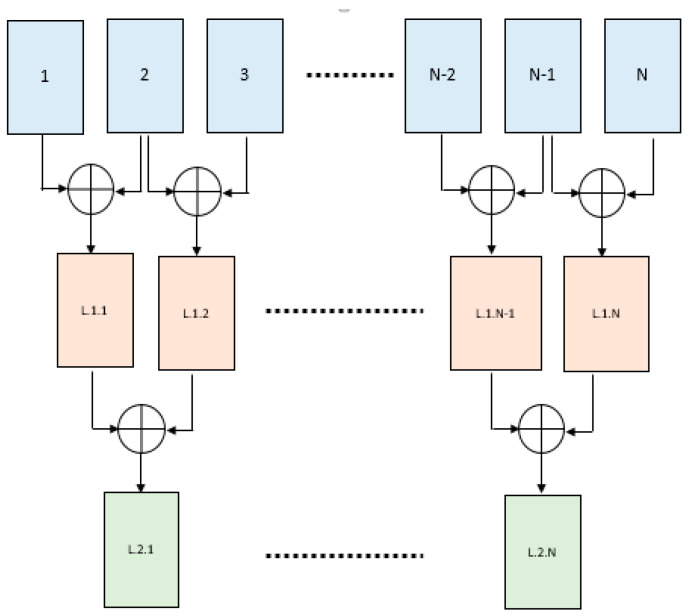
The Multi-Level Interframe Discrete Wavelet Transform (DWT). At the initial stage, the two successive frames are combined by taking their average. At level two, the frames from level one are averaged consecutively and this process continues.

The method employed for delivering a low-pass filtered video involved the utilization of a multi-level interframe discrete wavelet transform (DWT) with the Haar wavelet. Figure 12 depicts the operational principle of this transformation. During the initial stage, the two successive frames are combined by calculating their average. In the second level, the frames from the first level are averaged, and this process continues for subsequent levels. In this particular watermarking system, discrete wavelet transform (DWT) levels ranging from 12 to 16 are employed.

#### 5.1.2. Choice of Embedded Coefficients

**Figure 13:**
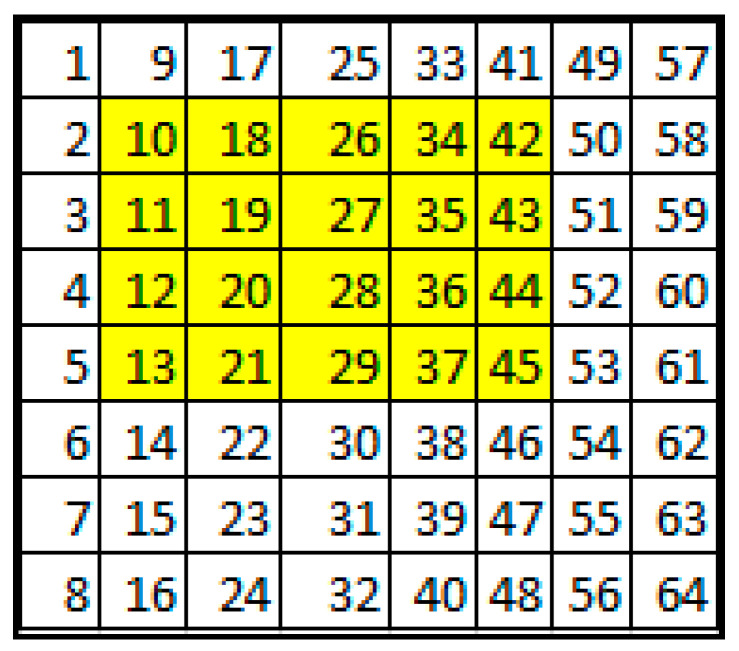
Selected coefficients of the DCT spectrum are presented in yellow.

In order to carry out the embedding process, certain coefficients from the discrete cosine transform (DCT) spectrum of the discrete wavelet transform (DWT)-filtered video sequence need to be chosen. Figure 13 displays the coefficients that meet the criteria for watermarking. Most of these items originate from the yellow region.

### 5.2. Investigation of Robustness with 3D Enhanced Hadamard Error-Correcting Code

The experiment utilized an HDTV video sequence with a resolution of 1080 × 1920 and a frame rate of 25 frames per second. The video was recorded with an AVCHD camera. The watermarking procedure was exclusively carried out on the luminance channel, following the conversion of RGB into the YCrCb color space. This choice was made due to the superior resilience of the luminance channel against distortions compared to other channels. An investigation was conducted to determine the number of embedded watermark bits that remain intact after compression attacks, without generating noticeable degradation. The deterioration of the watermarked output video was quantified using the SSIM (Structural Similarity) index. SSIM relies on the visual perception of the human eye, making it more effective in expressing distortion compared to established approaches such as PSNR (Peak Signal-to-Noise Ratio) or MSE (Mean Square Error) [23].

Here is the description of the coding procedure of watermarks for the luminance channel of HDTV video with a resolution of 1080 × 1920.

The encoder procedure starts after DWT filtering and DCT. The following steps have to be conducted:

1. Read 10 bits from the watermark. This determines the message word.
2. According to the Code Book, find the corresponding basic images and the associated codeword. The codeword in this case has a length of 512 bits.
3. Select 512 coefficients from DCT blocks.
4. Embed the codeword into the selected coefficients through QIM (Quantization Index Modulation).

The chosen error-correcting code is the Enhanced 3D Hadamard Code with dimensions of 8 × 8 × 8, resulting in a codeword length of 512 bits. According to the explanations in Chapter 2, the message code length is 10 bits. The Hadamard Code is denoted with the expression [codeword, message word, Hamming distance] and can be expressed as $\left[2^{m},m+1,2^{m-1}\right]$. The size of each DCT block is 8 × 8, and a total of 16 coefficients are taken from each block. Using this, it is simple to compute the total number of embedded watermark bits for each frame.

(28) $$E=\frac{H\cdot W}{B^{2}}\cdot \frac{M}{L}\cdot C=\frac{1920\cdot 1080}{8^{2}}\cdot \frac{10}{512}\cdot 16=10125\frac{Bit}{Frame}$$

The variables *H* and *W* represent the height and width of the frame, respectively. The letter B represents the size of the block in the DCT transform. The letter *M* represents the length of the message code. The letter *W* represents the length of the codeword in the 3D Hadamard Code, and the letter *C* represents the number of selected spectral coefficients.

Table 4 displays the findings of the capacity and robustness assessments. The compression assaults were executed using the H.264 codec, with various compression ratios. The approach successfully operates on the unprocessed video, resulting in an original data rate of 1.2 Gbit/s. A checkerboard pattern measuring 30 × 30 pixels was utilized as a watermark.

The watermarks were sequentially put into the frames. The Delta Quantization Index Modulation (QIM) determined the magnitude of the quantization intervals and was adjusted to a value of 11. The Delta value typically indicates the level of noise distortion present in the host video.

The video series was compressed using varying compression ratios. Even when compressed to 5 Mbit/s, which has a compression ratio of 1:240, it is still possible to recover all watermarks without any errors. The quality comparison between the originally compressed video and the embedded and compressed version reveals only a minimal change. The SSIM index for the video in this example is 98%.

### 5.3. An Analysis of the 3D Enhanced Hadamard Code Compared to the Reed–Solomon Code

A comparative analysis was conducted to demonstrate the efficacy of the Enhanced Hadamard Code (EHC) by comparing it with the widely recognized Reed–Solomon Code. The Reed–Solomon Code is widely recognized as an error-correcting code and is extensively utilized in many consumer electronics applications such as CDs, DVDs, Blu-rays, QR Codes, and data transmissions.

In order to achieve comparability between the Reed–Solomon Code and the Enhanced Hadamard Code, it is necessary to provide two parameters: the symbol length and the level of redundancy. The symbol length, also known as the block length, is equivalent to a 9-bit message. The 3D Enhanced Hadamard Code utilizes a Code Book in which each 9-bit message is assigned a 512-bit codeword represented by a cube. The Reed–Solomon Code utilizes a Code Book in which each 9-bit message is associated with a string of 4599 bits. This string consists of 512 symbols, with each symbol being 9 bits in length. The Reed–Solomon Code was designed to have a redundancy that allows for nearly the same number of correctable symbols as the Hadamard Code has correctable bits, which is n/2. The RS Code obtained is [511, 255], with a codeword length of n = 511 symbols and a message length of k = 255 symbols. Each symbol consists of 9 bits.

The performance of both algorithms is evaluated and documented in Table 5. With a data rate of 5Mbit/s and a compression ratio of 1:240, the EHC Code can successfully retrieve the complete watermark without any errors. In contrast, the RS Code exhibits a recovered watermark with a 17% error rate, indicating successful restoration.

At a transmission rate of 3 Mbit/s, the exceptional efficiency of EHC becomes even more evident. The EHC watermark has a margin of error with a precision of 1.7%. In contrast, the RS watermark is barely noticeable and has an error rate of 27.6%.

When considering capacity, it is important to note that the EHC code is superior to the RS code. This is because the EHC codeword length is significantly shorter, at 512 bits, compared to the RS codeword length of 4599 bits.

## 6. Conclusions

This study presents a novel kind of multidimensional Hadamard Error-Correcting Code, referred to as Enhanced Hadamard Error-Correcting Code (EHC), and its application in video watermarking. The implementation of this code in a video watermarking scheme provides compelling evidence of its efficacy.

The Enhanced Hadamard Code is significantly more efficient than the popular Reed–Solomon Code (RS Code). The watermarks of a video, which are safeguarded with EHC, have the ability to withstand a highly forceful compression attack, unlike with the RS Code.

Watermarks that are secured via EHC can be effortlessly retrieved without any errors from a video that has a compression ratio of 1:240. This compression ratio corresponds to a data rate of 5 Mbit/s.

If the embedding procedure utilizes the RS Code instead of EHC, it is not possible to achieve an error-free recovery of the watermarks. The error margin is approximately 17.5% and the watermark content is scarcely discernible.

The findings obtained are highly encouraging, demonstrating the significant potential of the new Enhanced Hadamard Code in the field of video watermarking. The Enhanced Hadamard Code (EHC) has the potential to boost the performance of light communication, particularly in the study field of wireless sensor network (WSN) and visible light communication (VLC) applications.

## Figures and Tables

**Table 1:** Code Book of [8,4,4] Hadamard Code.

Message Word					Hadamard Vector							
0	0	0	0		1	1	1	1	1	1	1	1
0	0	0	1		1	−1	1	−1	1	−1	1	−1
0	0	1	0		1	1	−1	−1	1	1	−1	−1
0	0	1	1		1	−1	−1	1	1	−1	−1	1
0	1	0	0		1	1	1	1	−1	−1	−1	−1
0	1	0	1		1	−1	1	−1	−1	1	−1	1
0	1	1	0		1	1	−1	−1	−1	−1	1	1
0	1	1	1		1	−1	−1	1	−1	1	1	−1
1	0	0	0		−1	−1	−1	−1	−1	−1	−1	−1
1	0	0	1		−1	1	−1	1	−1	1	−1	1
1	0	1	0		−1	−1	1	1	−1	−1	1	1
1	0	1	1		−1	1	1	−1	− 1	1	1	−1
1	1	0	0		−1	−1	−1	−1	1	1	1	1
1	1	0	1		−1	1	−1	1	1	−1	1	−1
1	1	1	0		−1	−1	1	1	1	1	−1	−1
1	1	1	1		−1	1	1	−1	1	−1	−1	1

**Table 2:** Code Book of [8,4,4] half-mode Hadamard Code.

Message				Hadamard Vector									Codeword							
0	0	0		1	1	1	1	1	1	1	1		1	1	1	1	1	1	1	1
0	0	1		1	−1	1	−1	1	−1	1	−1		1	0	1	0	1	0	1	0
0	1	0		1	1	−1	−1	1	1	−1	−1		1	1	0	0	1	1	0	0
0	1	1		1	−1	−1	1	1	−1	−1	1		1	0	0	1	1	0	0	1
1	0	0		1	1	1	1	−1	−1	−1	−1		1	1	1	1	0	0	0	0
1	0	1		1	−1	1	−1	−1	1	−1	1		1	0	1	0	0	1	0	1
1	1	0		1	1	−1	−1	−1	−1	1	1		1	1	0	0	0	0	1	1
1	1	1		1	−1	−1	1	−1	1	1	−1		1	0	0	1	0	1	1	0

**Table 3:** Part of the Code Book for [16,5,8] with Basis Images (cf. [17,18]).

Message	Basis Image	Matrix Element	Codeword
1 0 0 0 0	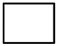	S(1,1)	0000000000000000
1 0 0 0 1	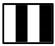	S(1,2)	0101010101010101
1 0 0 1 0	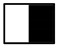	S(1,3)	0011001100110011
1 0 0 1 1	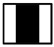	S(1,4)	0000111111110000
1 0 1 0 0	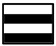	S(2,1)	0000111100001111
1 0 1 0 1	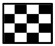	S(2,2)	0101101001011010
1 0 1 1 0	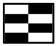	S(2,3)	0011110000111100
1 0 1 1 1	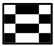	S(2,4)	0110100101101001
1 1 0 0 0	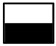	S(3,1)	0000000011111111
1 1 0 0 1	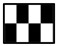	S(3,2)	0101010110101010
1 1 0 1 0	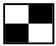	S(3,3)	0011001111001100
1 1 0 1 1	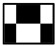	S(3,4)	0110011010011001
1 1 1 0 0	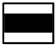	S(4,1)	0000111111110000
1 1 1 0 1	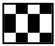	S(4,2)	0101101010100101
1 1 1 1 0	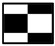	S(4,3)	0011110011000011
1 1 1 1 1	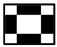	S(4,4)	0110100110010110

**Table 4:** Results of watermark measurements.

Data Rate	Compression Ratio	DWT Levels	Delta QIM	Extracted Watermark	SSIM Watermarking	SSIM Video
6 Mbit/s	1:200	16	11	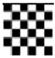	1	0.95
5 Mbit/s	1:240	16	11	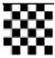	1	0.95
4 Mbit/s	1:300	16	11	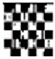	0.92	0.93
3 Mbit/s	1:400	16	11	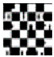	0.89	0.91
2 Mbit/s	1:600	16	11	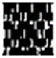	0.56	0.90

**Table 5:** Comparison of Enhanced Hadamard Code with Reed–Solomon Code.

Data Rate	EHC	Error %	Reed–Solomon	Error %
5 Mbit/s	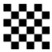	0	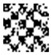	17.5
4 Mbit/s	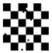	1.7	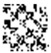	27.6
3 Mbit/s	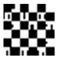	6.8	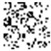	35.3
2 Mbit/s	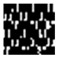	41.2	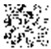	40.8

## Data Availability

Data is contained within the article.
